# The Neural Plasticity Theory of Depression: Assessing the Roles of Adult Neurogenesis and PSA-NCAM within the Hippocampus

**DOI:** 10.1155/2013/805497

**Published:** 2013-04-09

**Authors:** Steven R. Wainwright, Liisa A. M. Galea

**Affiliations:** Program in Neuroscience, Centre for Brain Health, Department of Psychology, University of British Columbia, Vancouver, BC, Canada V6T 1Z4

## Abstract

Depression is a devastating and prevalent disease, with profound effects on neural structure and function; however the etiology and neuropathology of depression remain poorly understood. Though antidepressant drugs exist, they are not ideal, as only a segment of patients are effectively treated, therapeutic onset is delayed, and the exact mechanism of these drugs remains to be elucidated. Several theories of depression do exist, including modulation of monoaminergic neurotransmission, alterations in neurotrophic factors, and the upregulation of adult hippocampal neurogenesis, and are briefly mentioned in the review. However none of these theories sufficiently explains the pathology and treatment of depression unto itself. Recently, neural plasticity theories of depression have postulated that multiple aspects of brain plasticity, beyond neurogenesis, may bridge the prevailing theories. The term “neural plasticity” encompasses an array of mechanisms, from the birth, survival, migration, and integration of new neurons to neurite outgrowth, synaptogenesis, and the modulation of mature synapses. This review critically assesses the role of adult hippocampal neurogenesis and the cell adhesion molecule, PSA-NCAM (which is known to be involved in many facets of neural plasticity), in depression and antidepressant treatment.

## 1. Neural Plasticity and Disease

As the fundamental units of the brain, neurons function to integrate and transmit a myriad of signals across vast and complex networks. Neurons by definition are continually forming, eliminating, and modulating (strengthening and weakening) connections in response to the constant flow of information. In mediating and responding to activity, neurons, including their processes and synapses, must be plastic. Thus neural plasticity may then be defined as the ability of neurons and neural elements to adapt in response to intrinsic and extrinsic signals. As such, our ability to process and synthesize information, ultimately producing behavior, is dependent upon this neural plasticity [1]. It is therefore not surprising that dysregulation or disruption of neural plasticity is associated with neuropsychiatric and neurodegenerative disease.

Dynamic processes such as adult neurogenesis, the development of dendritic spines, and synaptic adaptations are included under the umbrella of neural plasticity and are essential to normal functioning. Aberrant neural production, connectivity, or transmission is invariably present under disease states, such as Alzheimer's disease, schizophrenia, or depression [2–5]. Each aspect of neural plasticity can independently and additively cause or contribute to the disease state. Thus, it is of fundamental importance to elucidate the neurological underpinnings of these disease states at the cellular level, in order to understand etiology and better develop effective treatment. 

## 2. Adult Hippocampal Neurogenesis

Neural stem cells were first identified in the adult brain of rodents more than 50 years ago [6] and are found across a variety of species including humans [7]. Interestingly, the production of new neurons is typically limited to two regions: the subventricular zone, which lines the lateral ventricles and sends new neurons to the olfactory bulb via the rostral migratory stream, and the subgranular zone of the hippocampus [8].

It is important to note that neurogenesis, as defined here, requires the proliferation, survival, and differentiation of newly generated cells into neurons. Any number of internal and/or external factors may independently affect the proliferation of progenitor cells, their differentiation into neurons, or their survival rates [9–14]. Thus merely identifying cells as having been produced in the adult brain, without further demonstrating that they survive and become neurons, is insufficient to conclude that neurogenesis has occurred.

Although new neurons can be labelled and observed, the exact role of these neurons in the function of the hippocampus remains to be fully elucidated. New neurons produced in the subgranular zone have been associated with a number of functions [15, 16]. There is solid evidence that new hippocampal neurons are selectively and imperatively involved in spatial learning and memory [17–20]. New neurons in the hippocampus, perhaps more ambiguously, have also been associated with the etiology and treatment of depression [21–23]. Interestingly, the hippocampus is functionally dissociated along the dorsal-ventral axis, wherein the dorsal region plays a larger role in cognitive faculties, while the ventral region is more involved in emotionality [24, 25]. For the purpose this review, however, we will concentrate on the functions of the hippocampus associated with emotionality and stress and refer the reader to reviews that cover the hippocampus' cognitive associations (See: [26, 27]).

## 3. Cell Adhesion Molecules

Neural plasticity is a complex and varied process; from neural development, the migration of newly generated neurons, dendritic modifications, and synaptic modulation, many proteins facilitate and contribute to the malleability of neural tissue. Cell adhesion molecules (CAMs) are specialized proteins—typically expressed at the cell surface—which are important in synaptic function, synaptic plasticity, and remodeling of neural circuits [28]. The structure and function of CAMs vary widely within the nervous system, and the categorization and function of each CAM protein involved in neural plasticity are beyond the scope of this review (for a more detailed overview see [28, 29]). This review will instead focus on the potential role of a single CAM, the polysialylated form of neural cell adhesion molecule (PSA-NCAM), in the etiology and treatment of depression. 

 The neural cell adhesion molecule (NCAM) is a member of the immunoglobulin superfamily of cell adhesion molecules and serves to mediate Ca2^+^-independent cell-cell and cell-extracellular matrix (ECM) interactions [30]. Through homo- and heterophilic interactions, NCAM functions in cell migration, neurite outgrowth and targeting, axonal branching, synaptogenesis, and synaptic plasticity [30–32]. Neural plasticity mediated through the NCAM protein is facilitated through posttranslational modifications, the most important and prevalent of which is glycosylation with polysialic acid (PSA) [33]. Polysialic acid is a linear homopolymer of *α*2,8-linked sialic acid, which bears a negative charge, acting to abate NCAM-NCAM interactions and therefore interfere with cell adhesion [34]. PSA-NCAM serves to regulate cell-cell and cell-ECM interactions during times of plasticity.

In the adult brain PSA-NCAM is expressed on the cell surface of newly generated daughter cells, on neurites during outgrowth and path finding, and at the synapse of mature neurons [30]. The addition of the PSA moiety to NCAM is essential to neural remodelling and synaptic plasticity [10, 35, 36]. Selective cleavage of PSA in the adult brain inhibits activity-induced synaptic plasticity (induction of long-term potentiation (LTP) and long-term depression (LTD)) and alters the normal migration and integration of newly generated neurons within the hippocampus [10, 35], importantly though cleavage of PSA from NCAM does not disrupt normal basal synaptic neurotransmission or alter normal levels of neural proliferation or survival [10, 35]. PSA-NCAM is therefore a particularly interesting protein in that it is one that mediates plasticity at multiple levels, from neural proliferation, integration, differentiation, neuritic outgrowth, synaptogenesis, and modulation of mature synapses. 

## 4. Depression

Depression is a devastating neuropsychiatric disease that is both prevalent—with a lifetime incidence of 20% [37]—and costly, accruing over $83 billion (USD) annually in expense to society in the USA alone [38]. Depression is a spectrum disorder ranging from dysthymia to major depressive disorder (MDD), which also includes seasonal (seasonal affective disorder) and bipolar subtypes; however general symptomology is characterized by a depressed mood and/or anhedonia [39]. The DSM-IV criteria for diagnosis with MDD may include secondary symptoms such as fatigue, insomnia or hypersomnia, changes in body weight, and impaired cognition [39]. Although depression is prevalent and carries a large burden of disease, the pathoetiology of depression is poorly understood. While antidepressant drugs do exist, they are not ideal, as only a segment of patients is effectively treated, and the therapeutic onset is delayed [40–42]. Moreover, the exact mechanism of these drugs remains to be elucidated, though several theories do exist. The existing theories of etiology and treatment of depression will be briefly discussed with an aim of integrating and placing neurogenesis and PSA-NCAM related plasticity within the current state of knowledge.

## 5. Monoamine Theory of Depression

Much of the current understanding of depression has been built upon the serendipitous discovery of the mood-elevating effects of iproniazid, a monoamine oxidase inhibitor (MAOI), during clinical trials for antituberculosis agents in the early 1950s [43, 44]. Around the same time the first tricyclic antidepressant (TCA), imipramine, a drug also known to modulate monoaminergic neurotransmission, was discovered to relieve depressive symptoms [45]. Both compounds enhance monoaminergic neurotransmitters (primarily serotonin and norepinephrine) function in the synapse and therefore acted as a springboard for the rational design of drugs which specifically enhance the transmission of these neuromodulators. Further, these findings resulted in the development of the monoamine hypothesis of depression [46, 47]. The monoamine hypothesis of depression postulates that the pathophysiological basis of depression is due to the deficient activity of monoamines in the central nervous system [48]. 

 While first generation antidepressant drugs can indeed be effective, remission rates still typically remain below 60%, and treatment is associated with potentially severe side effects, causing a third of patients to discontinue treatment [49, 50]. Thus new drugs, such as selective serotonin reuptake inhibitors (SSRIs), have been developed in an attempt to enhance efficacy and reduce the side effects observed with the broader-acting first generation compounds. However, the ability of these newer drugs to alleviate depression is, unfortunately, typically lower than that of MAOIs and TCAs [50]. SSRIs are the primary first line treatment for patients with major depressive disorder (MDD), yet only a third of patients will respond to these drugs following initial treatment [41]. Even in patients that are initially responsive to treatment, up to 57% will have depressive symptoms return due to a loss of drug efficacy [40]. 

While it is known that antidepressants modify monoaminergic neurotransmission, it is not known how antidepressants exert their therapeutic effects, as the influence on monoamines occurs within hours of treatment, but alleviation of depressive symptoms requires weeks of exposure [51–54]. The monoamine theory explains this delayed efficacy of treatment as the time required for the 5-HT1A serotonin autoreceptor to sensitize and normalize serotonergic tone at the synapse [42, 55, 56]. However, disrupting or ablating portions of the serotonergic system fails to induce a depressive phenotype [57], suggesting that the dysregulation of serotonergic neurotransmission is not the only underlying factor in depression. Given the prevalence of treatment-resistant forms of depression and the limited long-term efficacy of current drugs, novel targets are needed for the development of more efficacious pharmacological treatments. 

## 6. Neurogenic Hypothesis of Depression

As noted earlier, neurogenesis in the adult hippocampus is seen across a variety of species, including humans [7]. Adult hippocampal neurogenesis has been hypothesized to play a potentially important role on the pathology and successful treatment of depression. A neurogenic hypothesis of depression has been postulated, which suggests that reduced adult hippocampal neurogenesis may underlie the pathoetiology of depression, while antidepressant efficacy depends on the upregulation of hippocampal neurogenesis [58]. 

## 7. Etiology of Depression and Animal Models

As with other neuropsychiatric disorders, depression has a multifaceted and varied etiology, including genetic, epigenetic, and environmental factors. The most prevalent of these factors is stress. Stress is cited as the leading cause of depression by depressed patients [59]. Unipolar depression is associated with abnormal hypothalamic-pituitary adrenal (HPA) axis function such as hypersecretion of cortisol, abnormal diurnal secretion of cortisol resulting in a flattened circadian rhythm and impaired negative feedback [59, 60]. Interestingly, normalization of HPA function is seen after chronic exposure to antidepressants, an effect coincident with, or slightly preceding, behavioural alleviation of depressive symptoms [61]. As such normalization of HPA function has become a major target of novel therapies [59, 62].

Many animal models of depression capitalize on the association of stress and depression; such models have a solid ethological basis. For instance, models using exposure to chronic variable stress (CVS) have shown good face, construct, and predictive validity, making it one of the most commonly used paradigms to model depression [63]. For example, CVS increases immobility in the forced swim test (FST, a putative measure of behavioural despair) [64], reduces sucrose preference (anhedonia) [65], increases novelty-induced hypophagia [21, 64], reduces hippocampal neurogenesis [21, 65], and reduces the expression of proteins associated with neuroplasticity such as PSA-NCAM [66]. Although it is not possible to model all symptoms of depression in rodents, a battery of key endophenotypes can be examined such as anhedonia, body weight changes, behavioural despair, HPA function, and brain alterations (such as reduced hippocampal volume, neurogenesis, and neural plasticity), as have been observed in human patients with depression [67].

## 8. Stress, Depression, and Reduced Neural Plasticity

The hippocampal formation is an area rich in mineralocorticoid (MRs) and glucocorticoid receptors (GRs). These receptors function in the maintenance of basal HPA tone and in the regulation of negative feedback of glucocorticoid release during a stress response [68]. Given this, it is not surprising that the hippocampus is particularly vulnerable to the effects of stress and depression [69, 70]. Depressed patients have reduced hippocampal volume which varies with the number of episodes and duration of the illness [71–73]. Similarly, postmortem studies of hippocampal tissue collected from depressed patients have shown alterations in gray matter density, reductions in neuropil, and decreased hippocampal neurogenesis [22, 23, 74–77]. Animal models of depression using exposure to chronic stress demonstrate the same changes in hippocampal structure and show reductions in neural plasticity, including reductions in neurogenesis and the expression of proteins associated with neural plasticity [21, 64, 65, 78]. Chronic stress exposure also reduces dendrite length and complexity [79, 80], spine density [81], and NCAM expression [65] and modifies expression of synaptic SNARE proteins [82] in the hippocampus. 

Stabilization of the HPA axis by antidepressants is associated with improved mood scores that either precedes or is coincident with the behavioural alleviation of depressive symptoms in humans [61]. Thus the improvements in negative feedback inhibition of the HPA axis that correspond to improvement in depressive symptoms with antidepressant treatment may be attributable to neural adaptation in limbic structures that regulate feedback inhibition of HPA axis such as the hippocampus. Interestingly chronic antidepressant treatment is seen to reverse the negative effects of stress on hippocampal volume [65, 83], though the exact contribution of neural plastic elements that underlie this enhancement is not fully understood.

## 9. Neurogenesis in Antidepressant Efficacy

The neurogenic hypothesis of depression is predicated not only on the previous findings that chronic stress decreases hippocampal neurogenesis [21, 65, 84] but also that antidepressant drugs can both prevent [83, 85] and reverse [21, 65, 86] this effect (see Table 1). The enhancement of adult hippocampal neurogenesis is dependent on chronic, but not acute, exposure to antidepressants, occurring in a manner that temporally coincides with the delayed clinical efficacy of these drugs [9, 21]. This delayed enhancement of neurogenesis is mediated through the increased proliferation of neural progenitor cells, while the proportion and the differentiation of surviving cells into neurons remain constant [9]. Several classes of antidepressant including SSRIs, TCAs, MAOIs, SNRIs, and melatonergic antidepressants increase neurogenesis in the hippocampus [87, 88]. Moreover, the nonpharmacological antidepressant treatment of electroconvulsive therapy (ECT) also enhances neurogenesis in the adult hippocampus [89, 90]. ECT is the most effective antidepressant treatment in depressed patients who are treatment resistant [91], and it can upregulate neurogenesis to levels nearly twice that of pharmacological antidepressants [89]. Similarly, recent human studies suggest that the broader acting, typically more efficacious, TCA and MAOI classes of antidepressant increase cell proliferation levels beyond that of SSRIs [23]; further indicating a role for hippocampal neurogenesis in successful antidepressant treatment.

While it is accepted that chronic antidepressant treatment upregulates hippocampal neurogenesis, some studies have suggested that increased neurogenesis is absolutely necessary for antidepressant efficacy. Specifically, if hippocampal neurogenesis is reduced via localized irradiation, antidepressants lose their behavioural efficacy in rodent models of depression [21]. However, use of the cytostatic agent methylazoxymethanol (MAM) to reduce hippocampal neurogenesis failed to disrupt the behavioural efficacy of chronic antidepressant treatment on sucrose preference or in the forced swim test; antidepressant treatment still increased hippocampal volume and NCAM expression, despite the disruption of hippocampal neurogenesis [65]. Nonetheless, neurogenesis does seem to be required for the attenuation of anxiety-like behaviour as measured in the novelty suppressed feeding (NSF) test, suggesting a role for neurogenesis in specific aspects of antidepressant efficacy. Indeed, further research has shown neurogenic-dependent and -independent effects of an antidepressant drug, where hippocampal neurogenesis is only required for the alleviation of some anxiety/depressive-like behaviour [64, 92–94]. These findings point toward multifaceted and pleiotropic effects of antidepressants on neural circuitry. Interestingly anxiolytic drugs, such as benzodiazepines, also show efficacy in the NSF test with acute treatment, while antidepressants require chronic, neurogenesis-enhancing treatment [95]. It is then possible that the reduction in glucocorticoids from anxiolytic treatment mediates the reduction in anxiety-like behaviour in the NSF test; therefore antidepressant-induced neurogenesis may mediate a similar effect on glucocorticoid levels. Indeed this may be the case, as hippocampal neurogenesis buffers the stress response and normalizes glucocorticoid release after stress [96]. Synder and colleagues found that inhibition of hippocampal neurogenesis, either transgenically or via irradiation, attenuated the recovery of basal HPA tone following a stressor and the normal suppression of glucocorticoid release during the dexamethasone suppression test [96]. As such, there is likely a role for hippocampal neurogenesis in reestablishing normal HPA tone and regulating a normal HPA response, possibly through GR-mediated negative feedback. Thus the effectiveness of antidepressant treatment and the importance of ensuing neurogenesis within different behavioural measures of antidepressant efficacy may represent the underlying mechanisms through which antidepressants are exerting their effects. Separate facets of antidepressant-induced neural plasticity may therefore facilitate drug action such as neurogenesis-mediated normalization of the stress response in the NSF test versus modulation of monoaminergic tone through synaptic plasticity in the sucrose preference test and FST. Hence the contribution of broader neural plasticity, beyond neurogenesis, is also of interest and has been proposed as the underlying mechanism of antidepressant efficacy [65].

## 10. Potential Role for PSA-NCAM in Antidepressant Efficacy

The expression of PSA-NCAM within the adult nervous system is essential in multiple facets of neural plasticity, including neurogenesis, synaptic plasticity, and neurite outgrowth [10, 30, 31, 97, 98]. Importantly, PSA-NCAM functions across all aspects of hippocampal neurogenesis (proliferation, migration, differentiation, and survival), therefore changes in the polysialylation of newly generated neurons alter neurogenesis as a whole [99–104] (see Table 2). For instance, chronic mild stress reduces the expression of both the core NCAM protein [65] and the addition of the PSA moiety [11, 105–108] in the hippocampus; however these changes are dependent on the type of stressor [109] (see Table 3). Alterations in PSA-NCAM expression following stress are also seen in other regions associated with depression including the amygdala and prefrontal cortex [110–113]. Similarly, PSA-NCAM expression is reduced in amygdala of patients with major depressive disorder [2, 114]; however no change is seen in the PFC [115]. Conversely, chronic antidepressant treatment modulates PSA-NCAM expression throughout the limbic system in animal models of depression. [11, 116–119]. Moreover, antidepressant treatment reduces PSA-NCAM expression in the dorsal raphe nucleus [116], implicating PSA-NCAM in antidepressant-induced plasticity related to serotonergic neurotransmission. Taken together these findings suggest PSA-NCAM may play a fundamental role in mediating broad effects of antidepressant treatment across multiple forms of neural plasticity.

Previous studies have shown PSA-NCAM is essential in the activity-dependent induction of hippocampal LTP and LTD [35], where the polysialylation of NCAM is positively correlated with neural activity of glutamatergic neurons [120, 121]. Moreover, inhibiting the polysialylation of NCAM reduces the rate of dendritic spine formation and decreases the stability of these spines [122]. Interestingly PSA-NCAM is also directly related to monoaminergic neurotransmission, as PSA-NCAM expression is directly modulated by the 5-HT1A receptor [123]. Moreover, serotonergic innervation plays a role in regulating the expression of PSA-NCAM in the hippocampus [124]. Given the interaction of serotonergic neurotransmission with PSA-NCAM and its putative role in regulating synaptic plasticity, it is possible that PSA-NCAM may mediate some of the effects of antidepressant treatment on monoaminergic tone. 

The ability of PSA-NCAM to directly alter hippocampal neurogenesis is evidenced by the selective removal of the PSA moiety from NCAM with the bacteriophage enzyme EndoN. Application of EndoN disrupts normal migration of newly produced cells and alters differentiation of these new cells by shifting them toward a neuronal phenotype [10]. Selective cleavage of PSA also produces enhanced dendritic arborization, aberrant mossy fiber sprouting, produces ectopic synaptic bouton formation, and increases cell death within the hippocampus [97, 98]. 

Given the role of PSA-NCAM in mediating multiple aspects of neural plasticity it appears to function at the confluence of the prevailing theories of depression: monoaminergic, neurotrophic, and neurogenic, thus making it an interesting target for future study. Further research is necessary to elucidate the role of PSA-NCAM and similar proteins, in the etiology of depression and efficacy of antidepressant drugs. 

## 11. Role of Neurotrophic Factors in Antidepressant Efficacy

Brain-derived neurotrophic factor (BDNF) has been implicated in the etiology and treatment of depression. Depressed patients show decreased levels of serum BDNF [125, 126], which is correlated with decreased hippocampal volume and increased ratings of depression [126]. Conversely, increased BDNF levels are associated with antidepressant treatment and alleviation of depression [127]. These findings are mirrored in rodent models of depression [128–132] as chronic antidepressant treatment increases BDNF levels, coinciding with alleviation of depressive-like behaviours [130–132], and intracranial infusion of BDNF produces antidepressant effects [128, 133]. 

Activation of the TrkB neurotrophin receptor largely mediates the plasticity-enhancing actions of BDNF [134–136], where inhibition of TrkB signalling attenuates the behavioural efficacy of antidepressants [136]. Conversely BDNF, or more specifically proBDNF, via activation of the p75 neurotrophin receptor mediates plasticity-reducing actions such as apoptosis, neural atrophy, and synaptic pruning [137]. It has been postulated that the balance of TrkB and p75 signalling may underlie antidepressant effects of BDNF, suggesting a mechanism through which stress/depression may modulate the effects of BDNF. To this point, knocking out TrkB receptors fails to produce a depressive-like phenotype in neurogenesis-dependent or -independent behavioural measures of antidepressant efficacy [138, 139]; however sex differences exist as female TrkB knockouts do appear to develop a depressive-like behavioural phenotype [140]. 

Interestingly, PSA-NCAM interacts with BDNF, as removal of PSA from NCAM inhibits the induction of LTP, and the application of exogenous BDNF restores LTP at the affected synapse [141]. Further, disruption of the polysialylation of NCAM also disturbs the effects of BDNF on cortical neuron differentiation and survival, while the application of exogenous BDNF reverses these effects [142]. These findings suggest that PSA-NCAM may mediate the responsiveness of neurons to BDNF. It is known that PSA-NCAM interacts with, and may regulate, p75 expression in septal neurons [143] and newly generated neurons of the SVZ [144]. Indeed knockout of p75 significantly reduces the expression of PSA-NCAM in SVZ neuroblasts [145]. As previously mentioned the p75 receptor is involved in the regulation of adult hippocampal neurogenesis [146] and regulates neurogenesis stimulated by chronic antidepressant treatment [147]. Given the complex and varied role of BDNF in the etiology of depression and antidepressant treatment more research is needed to further elucidate a mechanism through which the modulation of BDNF exerts its influence. 

## 12. Gonadal Hormones, Depression, and Modulation of Neural Plasticity

Women are twice as likely as men to develop depression [148]. Sex differences are also seen in antidepressant efficacy as men have a better response to TCAs, while women have a better response to SSRIs [149], although these findings remain controversial. Any sex difference observed suggests that gonadal hormone levels are involved, and indeed there is evidence that androgens may protect males from the development of depression. Interestingly, there is an increased incidence of depression in males coinciding with the age related decline in testosterone levels [150–153]. Similarly, young hypogonadal males are more susceptible to developing depression [154], portending protective effects of testosterone against the development of depression. Testosterone has shown some antidepressant action as testosterone replacement therapies are efficacious in alleviating depressive symptoms in hypogonadal men [152, 155, 156]. Testosterone replacement also has efficacy as an adjunct treatment to clinical antidepressants in cases of treatment-resistant depression [155, 157]; however it should be noted that androgen therapies are not always seen to be effective for men suffering depression [158, 159].

 Gonadal hormones in females are also likely a factor in the treatment and etiology of depression. Times of dramatic hormone fluctuation, such as during the postpartum and perimenopause, are associated with an increased incidence in depression [160, 161]. In addition hormonal replacement can show antidepressant effects during the postpartum and in peri- and postmenopausal women [162–164]. Consistent with clinical studies, gonadal hormones are also effective as an adjunct therapy to chronic antidepressant treatment in animal models. For example, chronic imipramine increases hippocampal neurogenesis in intact but not in ovariectomized rats [165]. Another study found that an SSRI decreased immobility in the FST in ovariectomized female rats, but only with adjunct estradiol treatment [166]. Similarly, testosterone potentiates the effects of imipramine in increasing cell proliferation and facilitates the alleviation of depressive-like behavioural phenotypes in castrated socially isolated male rats [167]. Consequently androgens and estrogens may have antidepressant properties which could impede the development of, or ameliorate, extant depressive disorders.

Interestingly gonadal hormones modulate adult neurogenesis, BDNF levels, and PSA-NCAM expression in the hippocampus. Testosterone and its metabolite dihydrotestosterone (DHT) both serve to enhance hippocampal neurogenesis through improved cell survival via an androgen-dependent mechanism within the dentate gyrus [168]. Similarly, estrogens are able to increase adult neurogenesis via proliferation or survival depending on duration of treatment [169]. Estrogens regulate the polysialylation of NCAM across the estrous cycle [170], while the removal of testicular hormones decreases PSA-NCAM expression in the dentate gyrus [11]. Significant interplay exists between gonadal hormones and BDNF, as testosterone and DHT interact with BDNF to modulate synaptic plasticity, dendritic morphology, and neurogenesis in the central nervous system [171–174]. Estrogens and BDNF also share widespread interactions, as estradiol regulates hippocampal BDNF [175, 176] levels possibly through an estrogen-sensitive response element on the BDNF gene [177]. Conversely, BDNF mediates estradiol-induced alterations in hippocampal dendritic spine density [178]. Thus gonadal hormones may play a role in facilitating antidepressant efficacy through the modulation of neural plasticity. 

## 13. Effects of Experimental Manipulation on Neural Plasticity

Many of the studies examining a reduction in neurogenesis have used viral vectors, irradiation, or the cytostatic agent, methylazoxymethanol. It is important to note that the experimental manipulation of neurogenesis also likely produces downstream effects on neural plasticity in a much broader sense, including the expression of cell adhesion molecules (including PSA-NCAM), synaptic proteins, and BDNF. As such, it is difficult to know for sure whether the attenuation of antidepressant efficacy via reductions in neurogenesis is due to changes in neurogenesis levels or other aspects of neural plasticity. For instance, radiation levels as low as 2.5 Gy significantly reduce the density of BDNF-expressing neurons [179] and the expression of PSA-NCAM in the hippocampus [180]. This is important as a 10 Gy dose of radiation is typically used to inhibit neurogenesis in studies assessing attenuated neurogenesis [21, 96, 181], suggesting BDNF and PSA-NCAM expression will also be affected. This suggests that the effects of radiation affect proteins associated with neural remodelling and synaptic plasticity as well as neurogenesis [180]. It is important to address these caveats in future research to further elucidate the role of neurogenesis and neural plasticity in depression and its treatment. 

## 14. Conclusion

Depression is a complex neuropsychiatric disease with a poorly defined etiology. While hosts of antidepressant drugs do exist, they are often inefficacious. Elucidating the neural underpinnings of depression and fully understanding the pleiotropic effects of current antidepressant compounds, beyond their role in modulating monoaminergic neurotransmission, are necessary for the development of more effective drugs. The potential role of neurogenesis, both its decline with the occurrence of depression and its enhancement by chronic treatment with antidepressant drugs and therapies, has provided a promising avenue for research. Though significant findings have been made relating neurogenesis to the effective treatment of depression, large gaps in our understanding still exist. To this end, the role of synaptic proteins and cell adhesion molecules, including PSA-NCAM, in the etiology and treatment of depression should also be investigated. As with neurogenesis, PSA-NCAM is reduced in depressed patients and in models of depression, while chronic antidepressant treatment increases expression of PSA-NCAM. Importantly however, PSA-NCAM mediates multiple facets of neural plasticity, including neurogenesis and synaptic plasticity, in addition to mediating effects of neurotrophic factors, such as BDNF. PSA-NCAM therefore functions at the confluence of many forms of neural plasticity, and its mechanisms bridge several theories of depression: monoamine, neurogenic, and neurotrophic. This review has focused on the potential roles of neurogenesis and PSA-NCAM in depression; however many other proteins are associated with neural plasticity and depression [29, 182, 183]. Given the limitations in the understanding of the genesis of depression and current antidepressant treatments, continued research into the exact contribution of adult neurogenesis and the potential roles of proteins associated with neural plasticity and the continued perusal of a broader, neural plasticity hypothesis of depression are certainly warranted.

## Figures and Tables

**Table:** (a) Animal models of depression

Reference	Species	Sex	Model	Antidepressant	Method of ablation	Summary of findings
Snyder et al. 2011 [96]	Mouse	M	Restraint	—	X-ray; transgenic	Neurogenesis-dependent regulation of HPA response to stress and behavioural measures (NSF, FST, and SC)
David et al. 2009 [64]	Mouse	M and F	Chronic corticosterone administration	Fluoxetine	X-ray	Neurogenesis-dependent (NSF) and -independent (OFT, FST) aspects of antidepressant efficacy
Bessa et al. 2009 [65]	Rat	M	Chronic mild stress	Imipramine; fluoxetine	MAM	Neurogenesis-dependent (NSF) and -independent (SC, FST) aspects of antidepressant efficacy. Significant alterations in neural plasticity associated with antidepressant efficacy
Surget et al. 2008 [92]	Mouse	M	Chronic unpredictable stress	Imipramine; fluoxetine		Neurogenesis-dependent (NSF, CS, ST) and -independent (A) aspects of antidepressant efficacy
Holick et al. 2008 [93]	Mouse	M	—	Fluoxetine	X-ray	Neurogenesis-independent effects of antidepressant efficacy
Airan et al. 2007 [94]	Rat	F	Chronic mild stress	Imipramine; fluoxetine	X-ray	Neurogenesis-dependent (NSF) and -independent (OFT) aspects of antidepressant efficacy
Alonso et al. 2004 [85]	Mouse	M	Chronic mild stress	Fluoxetine	—	Decreased cell proliferation in the DG, while chronic fluoxetine blocked this effect
Santarelli et al. 2003 [21]	Mouse	M	—	Imipramine; fluoxetine	X-ray	Neurogenesis-dependent (NSF) antidepressant efficacy
Czéh et al. 2002 [78]	Rat	M	Social subordination	—	—	Decreased cell proliferation and survival in the DG
Malberg et al. 2000 [9]	Rat	M	—	Fluoxetine; reboxetine; tranylcypromine; ECS	—	Chronic, but not acute, treatment with monoaminergic antidepressant and ECS increased cell proliferation in the DG

**Table:** (b) Human studies of depression

Reference	Subjects	Sex	Population assessed	Antidepressant	Effect on neurogenesis
Cobb et al. 2013 [74]	Humans	M and F	Depressed patients postmortem	—	No significant difference in number of granule cells between depressed subjects and controls; decreased hippocampal volume correlating with duration of disease
Boldrini et al. 2013 [75]	Humans	M and F	Depressed patients postmortem	SSRIs; TCAs	Depression is associated with a decreased number of granule neurons, correlated with reduced DG volume. SSRI and TCA treatment increase granule neuron number and DG volume
Boldrini et al. 2012 [22]	Humans	M and F	Depressed patients postmortem	SSRIs; TCAs	Both antidepressant classes increase cell proliferation over untreated depressed patients and controls; NPCs associated with angiogenesis
Boldrini et al. 2009 [23]	Humans	M and F	Depressed patients postmortem	SSRIs; TCAs	Both antidepressant classes increase cell proliferation over untreated depressed patients and controls
Stockmeier et al. 2004 [76]	Humans	M and F	Depressed patients postmortem	—	Increased density of granule cells in the DG of depressed subjects compared to controls

NSF: novelty suppressed feeding; FST: forced swim test; SC: sucrose consumption; OFT: open field test; CS: coat state; ST: splash test; A: actimeter; MAM: methylazoxymethanol acetate (cytostatic agent).

**Table 2:** Selected publications delineating the role of PSA-NCAM in adult hippocampal neurogenesis.

Authors	Species	Role in neurogenesis	Ablation method	Findings
McCall et al. 2013 [97]	Rat	Neurite outgrowth; survival	EndoN	Cleavage of PSA-NCAM caused expanded dendritic arborization and increased cell death
Burgess et al. 2008 [10]	Rat	Migration; differentiation	EndoN	Cleavage of PSA-NCAM disrupts normal migration and differentiation of newly generated neurons in the DG
Seri et al. 2004 [99]	?	Differentiation	—	PSA-NCAM is highly expressed in the entire cell body and growing processes of D cells (precursors in the generation of new granule neurons in the dentate gyrus)
Ni Dhuill et al. 1999 [100]	Human	Proliferation; neurite outgrowth	—	Hippocampal expression of PSA-NCAM throughout life in humans closely resembles that of the rat. Expression largely contained to granule cells of the dentate gyrus and their mossy fiber axons, with large reductions in expression with age
Seki and Rutishauser 1998 [98]	Mouse	Neurite outgrowth	NCAM KO; EndoN	Aberrant collateral sprouting of mossy fibers and ectopic synaptic bouton formation
Kuhn et al. 1996 [101]	Rat	Migration	—	Age related decline in PSA-NCAM expression in the GCL, reduced migration of PSA-NCAM expressing cells into the GCL
Fox et al. 1995 [102]	Rat	Proliferation	—	PSA-NCAM expression decreases with age, coinciding with decreased cell proliferation
Seki and Arai 1993 [103]	Rat	Proliferation; migration; differentiation	—	Newly generated granule cells in the dentate gyrus express a highly polysialylated form of NCAM, involved in the migration of immature neurons from the subgranular zone into the GCL
Seki and Arai 1991 [104]	Rat	Proliferation	—	Highly polysialylated form of NCAM is persistently expressed in the adult dentate gyrus

EndoN: endoneuraminidase N; GCL: granule cell layer.

**Table:** (a) Animal models of stress and depression

Reference	Species	Sex	Model	Antidepressant	Effect on PSA-NCAM
Gilabert-Juan et al. 2012 [110]	Mouse	M	Chronic restraint stress	—	*↔* in mPFC
Djordjevic et al. 2012 [111]	Rat	M	Chronic social isolation	—	↑ in HPC; ↓ PFC
Djordjevic et al. 2012 [109]	Rat	M	Chronic social isolation	Fluoxetine	↑ in HPC, ↓ by Flx treatment
Djordjevic et al. 2012 [112]	Rat	M	Chronic social isolation	Fluoxetine	↑ in PFC; ↓ by Flx treatment (with stress)
Gilabert-Juan et al. 2011 [113]	Mouse	M	Chronic restraint stress	—	↓ in CeM; *↔* in BLA; *↔* Me
Wainwright et al. 2011 [11]	Rat	M	Unpredictable chronic mild stress	—	↓ in HPC
Homberg et al. 2011 [116]	Rat	M	—	Fluoxetine	↓ in dRN; *↔* in mPFC; ↑ AMYG (adolescent), ↓ AMYG (adult)
Varea et al. 2007 [117]	Rat	M	—	Fluoxetine	↑ in HPC (str. luc. only); ↓ in Me and BMA; *↔* in BLA
Sairanen et al. 2007 [118]	Rat	M	—	Imipramine	↑ in HPC; ↑ plPFC
Varea et al. 2007 [119]	Rat	M	—	Fluoxetine	↑ in mPFC (whole); ↑ ilPFC; *↔* plPFC
Cordero et al. 2005 [105]	Rat	M	Chronic restraint stress	—	↓ in CeM; ↓ Me
Nacher et al. 2004 [66]	Rat	M	Oral corticosterone administration	—	↓ in HPC
Nacher et al. 2004 [106]	Rat	M	Chronic restraint stress; oral corticosterone administration	—	↓ in piriform cortex (oral CORT); ↑ in piriform cortex (restraint)
Pham et al. 2003 [107]	Rat	M	Chronic restraint stress	—	↑ in HPC (3 weeks), *↔* in HPC (6 weeks)
Sandi et al. 2001 [108]	Rat	M	Chronic restraint stress	—	↑ in HPC

**Table:** (b) Human studies of depression

Reference	Subjects	Sex	Population assessed	Antidepressant	Effect on PSA-NCAM
Maheu et al. 2013 [114]	Human	?	Depressed patients postmortem	Specific classes not disclosed	↓ in BLA
Gilabert-Juan et al. 2012 [115]	Human	M and F	Depressed patients postmortem	Specific classes not disclosed	*↔* in dlPFC
Varea et al. 2012 [2]	Human	M and F	Depressed patients postmortem	Specific classes not disclosed	↓ in BLA; ↓ in BMA

Me: medial amygdala; CeM: centromedial amygdala; BMA: basomedial amygdala; dRN: dorsal raphe nucleus; str. luc.: stratum lucidum; plPFC: prelimbic cortex; ilPFC: infralimbic prefrontal cortex; HPC: hippocampus; AMYG: amygdala; mPFC: medial prefrontal cortex.
